# *Mycoplasma bovis* subverts autophagy to promote intracellular replication in bovine mammary epithelial cells cultured in vitro

**DOI:** 10.1186/s13567-021-01002-z

**Published:** 2021-10-14

**Authors:** Yang Liu, Zhaoju Deng, Siyu Xu, Gang Liu, Yushan Lin, Sohrab Khan, Jian Gao, Weijie Qu, John P. Kastelic, Bo Han

**Affiliations:** 1 Department of Clinical Veterinary Medicine, College of Veterinary Medicine, China Agricultural University, Beijing, 100193 China; 2 College of Veterinary Medicine, Yunnan Agricultural University, Kunming, 650201 Yunnan China; 3 Department of Production Animal Health, Faculty of Veterinary Medicine, University of Calgary, Calgary, AB T2N 4N1 Canada

**Keywords:** *Mycoplasma bovis*, bovine mammary epithelial cells, autophagy, lysosome, intracellular replication

## Abstract

*Mycoplasma* species are the smallest prokaryotes capable of self-replication. To investigate *Mycoplasma* induced autophagy in mammalian cells, *Mycoplasma bovis* (*M. bovis*) and bovine mammary epithelial cells (bMEC) were used in an in vitro infection model. Initially, intracellular *M. bovis* was enclosed within a membrane-like structure in bMEC, as viewed with transmission electron microscopy. In infected bMEC, increased LC3II was verified by Western blotting, RT-PCR and laser confocal microscopy, confirming autophagy at 1, 3 and 6 h post-infection (hpi), with a peak at 6 hpi. However, the *M. bovis-*induced autophagy flux was subsequently blocked. P62 degradation in infected bMEC was inhibited at 3, 6, 12 and 24 hpi, based on Western blotting and RT-PCR*.* Beclin1 expression decreased at 12 and 24 hpi. Furthermore, autophagosome maturation was subverted by *M. bovis*. Autophagosome acidification was inhibited by *M. bovis* infection, based on detection of mCherry-GFP-LC3 labeled autophagosomes; the decreases in protein levels of Lamp-2a indicate that the lysosomes were impaired by infection. In contrast, activation of autophagy (with rapamycin or HBSS) overcame the *M. bovis-*induced blockade in phagosome maturation by increasing delivery of *M. bovis* to the lysosome, with a concurrent decrease in intracellular *M. bovis* replication. In conclusion, although *M. bovis* infection induced autophagy in bMEC, the autophagy flux was subsequently impaired by inhibiting autophagosome maturation. Therefore, we conclude that *M. bovis* subverted autophagy to promote its intracellular replication in bMEC. These findings are the impetus for future studies to further characterize interactions between *M. bovis* and mammalian host cells.

**Supplementary Information:**

The online version contains supplementary material available at 10.1186/s13567-021-01002-z.

## Introduction

*Mycoplasma* species belong to the class *Mollicutes* and lack a cell wall [1, 2]. There are several important pathogenic *Mycoplasma* species with adverse effects on animals and humans [2, 3]. These organisms have small genomes and they rely on their hosts for nutrients, resulting in specialized host-microbe interactions, some of which are not well characterized [4].

Autophagy is a homeostatic and highly conserved survival mechanism in eukaryotic cells to deliver unnecessary cytosolic proteins, organelles, as well as invading pathogens, to lysosomes for degradation [5]. Autophagy can be categorized into 3 main types: microautophagy, macroautophagy and chaperone mediated autophagy. Macroautophagy, which is the best studied, has emerged as an important cellular factor in both innate and adaptive immunity. Xenophagy is often used to describe macroautophagy of microbial pathogens [4, 6, 7]. After invading host cells, microorganisms are often targeted by autophagy [5], with the protein LC3, diffusely distributed in the cytoplasm, linked to the targeted substrates. The C-terminal fragment of LC3 is cleaved by Atg4 to form LC3-I, activated by Atg7 (an E1-like enzyme), transferred to Atg3 (an E2-like enzyme), and finally modified into a membrane-bound form, LC3II. In general, polyubiquitinated invading pathogens are recognized by the autophagy receptor, P62, that delivers the targeted substrate to LC3II-bound membranes, facilitating autophagosome formation. Beclin1 was disinhibited, serving as a “molecular scaffold” to link autophagosomes to a lysosome when autophagy was initiated. Autophagosome-lysosome fusion represents the next step in degradation, in which hydrolases, e.g., lysosomal-associated membrane protein 2a (Lamp-2a), are activated by acidification. The final outcome is recycling of cytosolic material or containment of intracellular pathogens [8].

Several studies have verified the crucial role of autophagy in controlling infections by various bacterial pathogens. Furthermore, some of these pathogens have developed strategies to circumvent autophagy or to use it to establish replicative niches within various cell types [9, 10]. For example, autophagy benefits several bacterial pathogens, including *Salmonella *and *Legionella,* although it is an effective defense mechanism inhibiting survival of *Mycobacterium tuberculosis* in host cells [5, 11, 12]. For *Mycoplasma* species, which are the smallest prokaryotes capable of self-replication, autophagy has an important role in host–pathogen interactions. For example, *Mycoplasma ovipneumoniae* induced autophagy in macrophages in vitro [13]. Furthermore, autophagy had a critical role in stimulating the inflammatory response to *Mycoplasma pneumoniae* infections in mice [14]. However, the fate of *M. bovis* targeted by autophagy remains uncertain. This bacterium, a hard-to-eradicate ruminant pathogen, causes substantial losses in animal production [3, 15, 16]. Infections with *M. bovis* are usually chronic and difficult to treat [15]. For example, conventional antimicrobial therapy is not very effective for *M. bovis* mastitis [1, 15, 16]. Furthermore, knowledge gaps in understanding how *M. bovis* persists within mammalian cells contribute to a lack of effective control strategies. Based on previous reports, *M. bovis* can invade into various host cells, including epithelial or immune cells [17–20]. Therefore, we hypothesized that *M. bovis* subverts autophagy to promote its intracellular replication. In the present study, *M. bovis* and primary bovine mammary epithelial cells (bMEC) were used to establish an in vitro model to characterize autophagy in host cells and resulting implications for survival and replication of intracellular *Mycoplasma*.

## Materials and methods

### Statement of ethics

This study was conducted in accordance with the ethical guidelines of China Agricultural University (CAU; Beijing, China). Prior to the beginning of the study, ethical approval was granted by the Departmental Committee of the College of Veterinary Medicine, CAU.

### Antibody and reagents

Enhanced Cell Counting Kit-8 (CCK-8), Ad-mCherry-GFP-LC3B, Ad-GFP-LC3B, Bicinchoninic acid (BCA) protein assay kit, Lyso-Tracker red and radioimmunoprecipitation assay (RIPA) lysis buffer (Beyotime Biotechnology) were purchased from Beyotime (Shanghai, China). The PPLO broth was from BD Biosciences (San Jose, CA, USA), whereas horse serum, Fetal Bovine Serum (FBS), Dulbecco modified Eagle medium (DMEM) and Hank balanced salt solution with Ca^2+^ and Mg^2+^ (HBSS) were purchased from Hyclone (Logan, UT, USA). Penicillin G, streptomycin, gentamicin, amphotericin and bovine serum albumin (BSA) were from Coolaber (Beijing, China). Collagenase IV, 0.4 mg/mL DNAse I, 0.5 mg/mL hyaluronidase I-S, rapamycin and 3-methyladenine were from Sigma-Aldrich (St. Louis, MO, USA). Polyvinylidene difluoride membrane was from Millipore (Bedford, MA, USA). Coverslips, 4′, 6-Diamidine-2′-phenylindole dihydrochloride (DAPI) and Triton X-100 were all purchased from Solarbio (Beijing, China). An enhanced chemiluminescence (ECL) kit was obtained from Thermo Fisher Scientific Pierce (Rockford, IL, USA). Anti-p62 antibody and anti-glyceraldehyde 3-phosphate dehydrogenase (GAPDH) antibody were purchased from Abcam (Cambridge, MA, USA). Anti-LAMP2 antibody and anti-β-actin antibody were obtained from Beyotime. Anti-LC3B antibody, anti-Beclin1 antibody and anti-cytokeratin 18 (CK18) were from Proteintech (Chicago, IL, USA). Peroxidase-conjugated goat anti-mouse IgG were from ZSGB-BIO (Beijing, China). Goat anti-rabbit IgG were from Beyotime. Alexa fluor conjugated antibodies were purchased from Cell Signaling Technology (Danvers, MA, USA) and Freund incomplete adjuvant was from Sigma-Aldrich.

### *M. bovis* strain and growth conditions

*M. bovis* strain PG45 (ATCC 25,523) was purchased from the ATCC. For infection experiments, *M. bovis* were cultured in PPLO broth with 20% horse serum and penicillin (100 IU/L) in 5% CO_2_ at 37 °C for 72 h. The PPLO broth was prepared by dissolving 21 g of Difco PPLO medium (BD Biosciences) and 2.5 g of yeast extract (BD Biosciences) in 700 mL of ultrapure water, then autoclaving it at 121 °C for 30 min. *M. bovis *were collected by centrifugation (8000 × *g* for 40 min) and then washed with phosphate-buffered saline (PBS). The number of colony forming units was determined by performing ten-fold serial dilutions in PBS and subsequently spotting on PPLOA plates [18], prepared by supplementing PPLO broth with 20% horse serum and 0.75% agar. The bacteria were suspended in PBS to a cell density of 10^8^ colony-forming units per milliliter (CFU/mL), and the suspension was stored at −70 °C until use [21].

### Mouse anti-*M. bovis* serum preparation

BALB/c mice, 6–8 weeks old, were immunized with 1  ×  10^6^ CFU *M. bovis* strain PG45 mixed with an equal volume of Freund incomplete adjuvant and delivered by multipoint subcutaneous injections. Mice were immunized 3 times at 2-week intervals. At 2 weeks after the last immunization, mice were euthanized and blood was collected and incubated at 37 °C for 0.5 h, followed by incubation at 4 °C for 0.5 h. Serum from all mice were combined to form a common pool and stored at −20 °C.

### Cell culture

Primary bovine mammary gland epithelial cells were collected as described [22, 23] from lactating Holstein cows with clinically healthy udders (milk somatic cell count  <  10^5^ cells/mL). Briefly,  ~ 100 g of mammary gland tissue was obtained within 30 min after slaughter and transported to the laboratory at room temperature (23  ±  3 °C) in 500 mL Hank balanced salt solution with Ca^2+^ and Mg^2+^ (HBSS) supplemented with 500 μL penicillin G (100 mg/mL), 500 μL streptomycin (100 mg/mL), 500 μL gentamicin (100 mg/mL), and 500 μL amphotericin (5 mg/mL).

The following were conducted in a laminar flow hood under sterile conditions. Tissue was washed with HBSS and surface tissue excised and discarded. Interior tissue was minced into 1 cm^3^ pieces, followed by incubation for 1 h in 500 mL HBSS supplemented with 500 μL penicillin G (100 mg/mL), 500 μL streptomycin (100 mg/mL), 500 μL gentamicin (100 mg/mL), and 500 μL amphotericin (5 mg/mL) at 37 °C. Then, tissue blocks were further minced into 1–5 mm^3^ pieces that were rinsed several times with HBSS to remove milk and blood. These tissue blocks were transposed into 250 mL HBSS with final concentrations of 0.5 mg/mL collagenase IV, 0.4 mg/mL DNAse I, 0.5 mg/mL hyaluronidase I-S, penicillin G (100 μg/mL), streptomycin (100 μg/mL), gentamicin (100 μg/mL), and amphotericin (5 μg/mL). Digestion was performed for 3 h at 37 °C, with the liquid swirled every 10 min. Then, the suspension was filtered through sterile metal strainers (pore size  ~ 1 mm^2^) and the cells were subsequently collected by centrifugation for 10 min at 1000 *g*. Pellets were resuspended in 1/2 volume HBSS, filtered through sterile metal strainers (pore size  ~ 0.5 mm^2^), and centrifuged for 10 min at 1000 *g*. Pellets were re-suspended in ½ volume HBSS, filtered through sterile metal strainers (pore size  ~70 μm^2^), and centrifuged for 10 min at 1000 *g*. The precipitate was re-suspended in 1 L full medium with 10% FBS and 1 mL penicillin G (100 mg/mL), 1 mL streptomycin (100 mg/mL), and 1 mL amphotericin (5 mg/mL). Cells were purified by differential adhesion, as follows [22, 23]. Cells were transferred into a 150 T flask and incubated in 5% CO_2_ at 37 °C for 30 min to allow fibroblasts to attach. Epithelial cells were decanted and counted after staining by trypan blue. Cells were seeded in 25 T flasks at a concentration of 1  ×  10^5^ cells/mL for routine subculture. One passage was defined as one time subculture and purification was done in passages 1–3. The bMEC in passages 6, 7, 8 and 9 were stained with CK18, a marker of luminal epithelial cells [24, 25], and identified by fluorescence microscopy. Briefly, bMEC were seeded in 6 well-plates with coverslips. When cells reached 60–70% confluence, they were fixed in 4% paraformaldehyde at room temperature for 15 min. Then cells were washed twice in PBS, resuspended in 1 mL PBS, and incubated with anti-CK18 antibody (at a dilution of 1:200 in PBS) at 4 °C overnight (14–16 h). Cells were washed twice in PBS and resuspended in 1 mL PBS, and incubated with Alexa Fluor-conjugated secondary antibodies for 0.5 h at RT. Thereafter, cells were stained with DAPI and imaged with a Nikon A1 LFOV confocal microscope at laser wavelengths of 405 and 488 nm. The purity of bMEC was defined as the proportion of CK18-positive cells among total cells (stained by DAPI). A random selection of 5 fields per sample was analyzed, with 3 repeats conducted for cells in each passage.

### Cell infection and gentamicin protection assay

**Figure 1:**
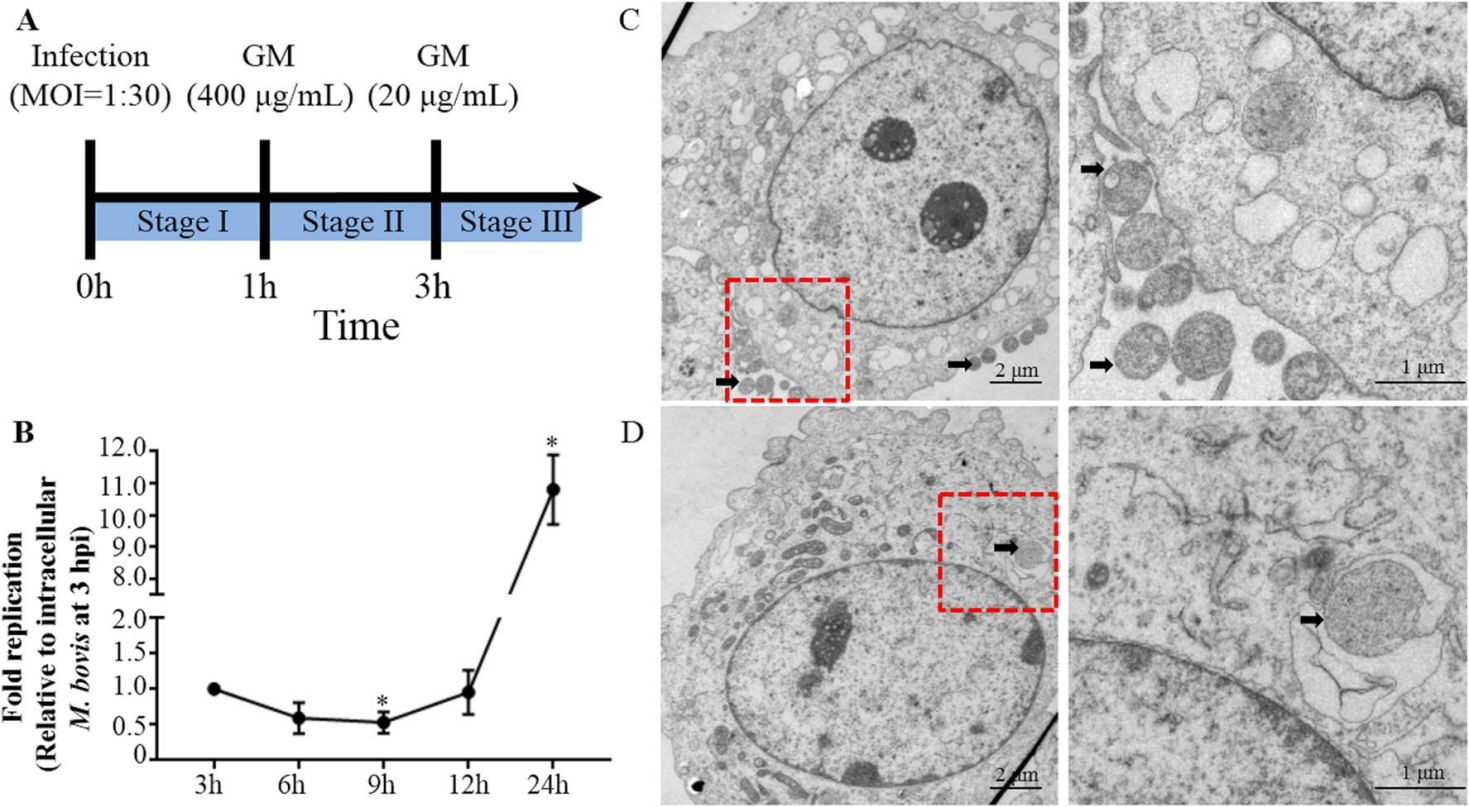
**Cell infection model constructed. A** Schematic overview. GM: gentamicin; stage I: bMEC infected (Time  =  0 h) with *M. bovis* inoculum at MOI of 1:30 for 1 h; stage II: extracellular *M. bovis* were killed by addition of 2 mL/well DMEM with 400 μg/mL gentamicin for 2 h at 37 °C; stage III: fresh DMEM with 10% fetal bovine serum (FBS) and 20 μg/mL gentamicin were added to the infected cells. MOI: multiplicity of infection. **B** Replication of intracellular *M. bovis* in the cell infection model. **C** Ultra-microstructure observation of adherent *M. bovis.* **D** Ultra-microstructure observation of intracellular *M. bovis.* Arrows point to *M. bovis*. Data are mean  ±  SD of 3 independent experiments. Standard deviations of individual measurements are indicated as bars. ^*^Compared to the control group (*p*  <  0.05).

The PG45 strain was used to explore intracellular replication of *Mycoplasma* in bMEC, following a protocol presented in Figure 1A. Viable *M. bovis* in the inoculum were counted, as described above. Specifically, cells were seeded at a concentration of 1  ×  10^5^ cells/mL in 6 well plates (2 mL per well) 24 h prior to experiments. The bMEC were inoculated (time  =  0 h) with *M. bovis* at multiplicities of infection (MOI) of 1:30 when cells reached 60–70% confluence. After infection at 37 °C for 1 h, the inoculum was removed and cells were washed twice with sterilized PBS, 2 mL/well, to remove nonadherent *M. bovis*. Thereafter, all extracellular *M. bovis* were killed by the addition of 2 mL/well DMEM with 400 μg/mL gentamicin for 2 h at 37 °C (time  =  1 h). Cells were again washed as described above. Finally, fresh DMEM with 10% fetal bovine serum (FBS) and 20 μg/mL gentamicin was added to the infected cells, 2 mL/well (time  =  1 h).

### Enumerating intracellular *M. bovis*

At designated time points, cells were washed thrice with PBS after treatment, as described above and the CFU were enumerated as described [18]. Cells were detached from plates with a 23-gauge needle and syringe; thereafter, bacterial concentrations were confirmed by plating ten-fold serial dilutions [18]. Fold changes in intracellular *M. bovis* loading were defined as the number of intracellular *M. bovis* at a subsequent time point, divided by the number of intracellular *M. bovis* at 3 hpi. The assay was repeated 3 times independently, with 3 biological replicates for each treatment. To enumerate CFU, *M. bovis* in each cell well were counted, with 6 repeats on plates.

### Transfection

The GFP-LC3B assay is used widely to monitor autophagy or colocalization with cargo [26, 27]. Ad-GFP-LC3B is an adenovirus expressing GFP-LC3B fusion protein, whereas Ad-mCherry-GFP-LC3B expresses mCherry-GFP-LC3B fusion protein [26]; both were used, in accordance with the manufacturer’s instructions, to transfect bMEC. Briefly, the bMEC were seeded on 6-well plates with coverslips, followed by incubation in 5% CO_2_ at 37 °C for 12 h. When bMEC density was 40–50% confluence, cells were infected with Ad-mCherry-GFP-LC3B or Ad-GFP-LC3B at MOI of 1:10 in DMEM containing 10% FBS. Then, cells were incubated in 5% CO_2_ at 37 °C for 24 h. Subsequently, these cells were used in infection experiments. Ad-mCherry-GFP-LC3B was used to demonstrate blockage of the autophagic flux in eukaryocytes [26]. Infection of Ad-mCherry-GFP-LC3B resulted in overexpression of tandem mRFP/mCherry-GFP proteins. In cells lacking an autophagic state, mCherry-GFP-LC3B was present in the cytoplasm as diffuse yellow fluorescence (combined effects of mCherry and GFP). However, in infected cells under an autophagic state, mCherry-GFP-LC3B targeted the autophagic substrate to form mRFP/mCherry puncta. The GFP is sensitive to low pH and the GFP signal is quenched in an acidic or mature autophagosome. The mCherry is stable when exposed to an acidic environment; therefore, quenching the GFP signal causes an LC3B puncta with mCherry signal only. When imaged under confocal microscopy, the mCherry-GFP-LC3B puncta will yield a red shift signal, indicating the autophagy flux was complete. However, if the autophagy flux is blocked, autophagosome maturation is inhibited and mCherry-GFP-LC3B produces yellow puncta. The bMEC were scanned with a Nikon A1 LFOV confocal microscope and numbers of RFP and GFP puncta were calculated [26, 28]. Twenty cells for each sample and at least 60 cells in each group were used for statistical analyses.

### Lyso-tracker red staining

Lyso-Tracker Red staining was used to detect acidification of lysosomes or mature autophagosomes [26]. Lyso-Tracker Red is based on DND-99, which is sensitive to a low pH. Lysosomes or mature autophagosomes marked by Lyso-Tracker Red yield red structures when acidification is activated. At designated time points, bMEC were stained with Lyso-Tracker Red. Briefly, cells were incubated with 50 nM Lyso-Tracker Red at 37 °C for 30 min and the fluorescence signal of Lyso-Tracker Red was observed at 0, 1, 3, 6, 9, 12, and 24 hpi with a Nikon A1 LFOV confocal microscope. Randomly selected 5 fields per sample were analyzed for each sample and 3 repeats were conducted.

### Induction and inhibition of autophagy

Following infection as described above, autophagy was induced by treatment with 20 μg/mL rapamycin or Hanks’ balanced salt solution (HBSS). Alternatively, autophagy was inhibited by pre-incubating bMEC with 5 mM 3-methyladenine (3-Ma) in full-nutrition medium for 3 h prior to infection. Moreover, effects of rapamycin and 3-Ma on *M. bovis* viability were determined. Specifically, 1 × 10^6^ CFU/mL *M. bovis* was incubated in PPLO medium with 20% horse serum, with or without 1 of the 2 compounds. The CFU of *M. bovis* was determined as described above, after incubation in 5% CO_2_ at 37 °C for 24 h. Effects of rapamycin or 3-Ma on viability of bMEC was evaluated with CCK-8 assays [27]. Briefly, bMEC were seeded in 96-well plates (1  ×  10^4^ cells/well) in 100 μL DMEM with 10% FBS. After incubation in 5% CO_2_ at 37 °C for 24 h, cells reached 60–70% confluence. Cells were treated with rapamycin (20 μg/mL in DMEM with 10% FBS), or 3-Ma (5 mM in DMEM with 10% FBS) or nothing (Control), followed by 24 h incubation in 5% CO_2_ at 37 °C. Then, fresh DMEM containing 10% CCK-8 was placed in the well. The cells were incubated in 5% CO_2_ at 37 °C for 2 h and optical density was determined with a microplate reader (Bio-Rad, Hercules, CA, USA) at 450 nm. The density of treated cells relative to control cells was calculated.

### Western blotting

At designated time points, cells were washed 3 times with PBS and total protein extracted with RIPA lysis buffer on ice. The liquid was centrifuged at 12 000 × *g* for 15 min. Protein concentrations were determined with a BCA protein assay kit, according to the manufacturer’s instructions. For each sample, equal amounts of protein were separated by SDS-PAGE. Subsequently, proteins were transferred onto a PVDF using a semidry blotting system. Blots were first blocked with 5% skim milk in Tween-20/TBS (TBST) at room temperature for 2 h, followed by 3 washes in TBST for 10 min each; then, membranes were incubated with each specific primary antibody overnight at 4 °C. After 3 washes in TBST for 10 min each, membranes were incubated with secondary antibodies for 1 h at room temperature (RT). Signals were detected using an ECL-Plus Western blot detection system and band density analyzed with Image J (National Institutes of Health, Bethesda, MD, USA).

### RNA extraction, cDNA synthesis and real-time PCR

At the various time points indicated, bMEC were harvested with 1 mL TransZol Up lysis solution (TransGen Biotech, Beijing, China). Total RNA was extracted with a total RNA extraction kit (TransGen Biotech), according to the manufacturer’s instructions. The cDNA was synthesized using TransScript^®^ II All-in-One First-Strand cDNA Synthesis SuperMix for PCR (TransGen Biotech). Both RNA and cDNA were quantified with a NanoDrop One spectrophotometer (Thermo Fisher Scientific, Waltham, MA, USA). For autophagy-associated genes, mRNA expression levels were verified with real time PCR. This assay included the following steps: pre-denaturation at 94 °C for 2 min, followed by 40 cycles of denaturation at 95 °C for 5 s and annealing at 60 °C for 60 s using the Applied Biosystems StepOnePlus Real Time PCR system (Thermo Fisher Scientific). For melt curve analysis, PCR products were heated from 55 to 95 °C, with the fluorescence signal checked every 0.5 °C to verify the specificity of each pair of primers. Cycle threshold (Ct) values were determined with StepOne TM Software version 2.3 (Thermo Fisher Scientific). In this study, ΔCt  =  Ct target gene − Ct endogenous control (arithmetic mean of the reference gene), whereas ΔΔCt  =  ΔCt sample − Ct control (uninfected cells). To visualize impacts of *M. bovis* on the responses of target genes in bMEC, relative mRNA expression data were presented as 2^−ΔΔCt^. Real-time quantitative PCR was used to amplify 100 ng of cDNA using the following pairs of primers: cattle LC3B upstream primer 5′-atgccgtccgagaaaacctt-3′ and downstream primer 5′-ccgggattttggtaggatgc-3′; cattle P62 upstream primer 5′-tctgccctgactacgaccta-3′ and downstream primer 5′-ccatgtttcagcttccggag-3′; cattle Beclin1 upstream primer 5′-gacactcagctcaacgtcac-3′ and downstream primer 5′-gcttcctcctgatccaacct-3′; cattle Lamp2a upstream primer 5′-ccgtgtctggagcatttcag-3′ and downstream primer 5′-ggtgtcatcatccagcgaac-3′; cattle GAPDH upstream primer 5′-attgaccttcactacatggt-3′ and downstream primer 5′-acccttcaagtgagccccag-3′. For these studies, GAPDH was the reference gene.

### Transmission electron microscopy

The bMEC were digested with trypsin and centrifuged at 1000 × *g* for 5 min. The cells were washed twice with PBS, fixed with 2.5% glutaraldehyde for at least 2 h, then fixed in 1% osmium tetroxide for 2 h at 4 °C. After dehydration in a graded ethanol series, samples were embedded in epoxy resin-acetone mixtures for 2 h, followed by immersion in a pure resin solution overnight at 37 °C. After polymerization, ultrathin sections were cut, stained with saturated uranyl acetate in 50% ethanol and lead citrate, and examined with a transmission electron microscope (JEM-1400, JEOL, Tokyo, Japan).

### Immunofluorescence staining and fluorescence microscopy

To assess co-localization of autophagy-associated genes with intracellular *M. bovis*, LC3B, Lamp-2a or *M. bovis* were detected by immunofluorescence staining and confocal laser microscopy [29]. Cells on coverslips were fixed with 4% paraformaldehyde (PFA) for 15 min at RT, then washed twice with PBS. Fixed cells were blocked with 3% bovine serum albumin (BSA) in PBS for 15 min, followed by permeabilization with 0.2% Triton X-100 in PBS for 45 min at room temperature. Afterwards, cells were incubated with an appropriate antibody for 1 h at room temperature, washed 3 times with PBS, then incubated with Alexa Fluor-conjugated secondary antibodies for 0.5 h at RT. Finally, coverslips were stained with fluorescence mounting medium containing DAPI and mounted on glass slides. Images were captured with a Nikon A1 LFOV confocal microscope at laser wavelengths of 405, 561 and 488 nm and analyzed with Image J software with the JaCoP plugin. Representative cells were selected and photographed. Twenty cells for each sample and at least 60 cells in each group were used for statistical analyses.

### Statistical analyses

All assays were repeated 3 times independently, unless otherwise stated. There were 3 biological replicates in every treatment, unless otherwise stated. All data were analyzed by 1-way ANOVA using SPSS 22.0 (IBM Corp., Armonk, NY, USA) and data reported as mean ± standard deviation of 3 independent experiments.

## Results

### *M. bovis* survival patterns in infected bMEC

The bMEC were identified by CK18 staining. The proportion of CK18-positive cells was  >  95% in cells from passages 6–9 (Additional file 1). There was no significant difference between the purity of bMEC in passage 6 versus passages 7–9 (Additional file 1, *p*  <  0.05) and bMEC from passages 6–8 were used for experiments. When bMEC infected by *M. bovis* were assessed by TEM at 1 hpi, some *M. bovis* were adherent on the surface of bMEC (Figure 1C), whereas intracellular *M. bovis* were in a membrane-like structure (Figure 1D). The intracellular *M. bovis* load in bMEC at 9 hpi decreased (*p*  <  0.05) to approximately half that at 3 hpi, although intracellular *M. bovis* increased at 24 hpi (Figure 1B; *p*  <  0.05).

### *M. bovis* induced autophagy in bMEC

**Figure 2:**
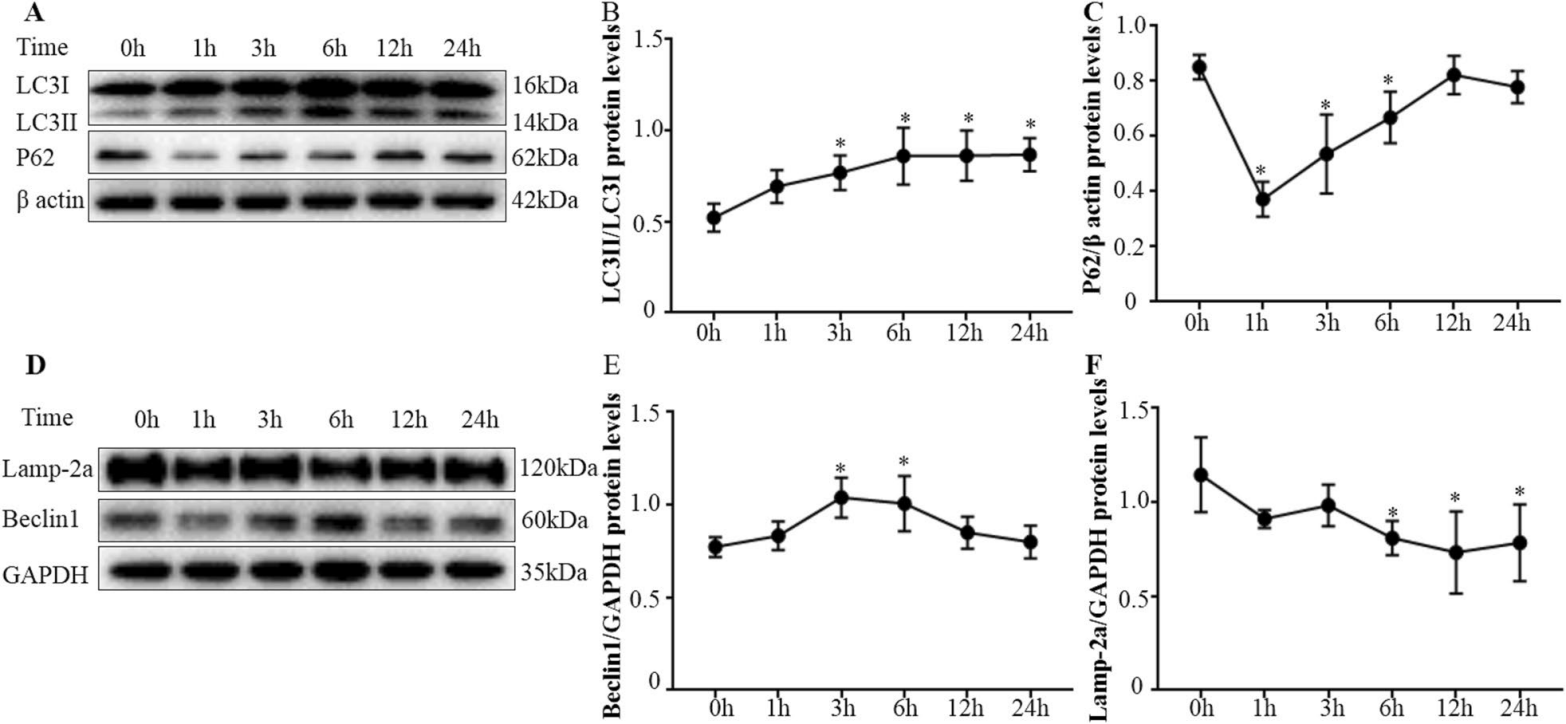
**Western blotting assays for protein levels of autophagy associated genes in bMEC infected by** *M. bovis.* **A** Representative Western blotting images of LC3, P62 and β actin. **B** Quantification of LC3 conversion (LC3I to LC3II). **C** Quantification of P62 expression. **D** Representative Western blotting images of Lamp-2a, Beclin1 and GAPDH. **E** Quantification of protein levels of Beclin1. **F** Quantification of protein levels of Lamp-2a. Data are mean  ±  SD of 3 independent experiments. Standard deviations of individual measurements are indicated as bars. ^*^Compared to the control group (*p*  <  0.05).

**Figure 3:**
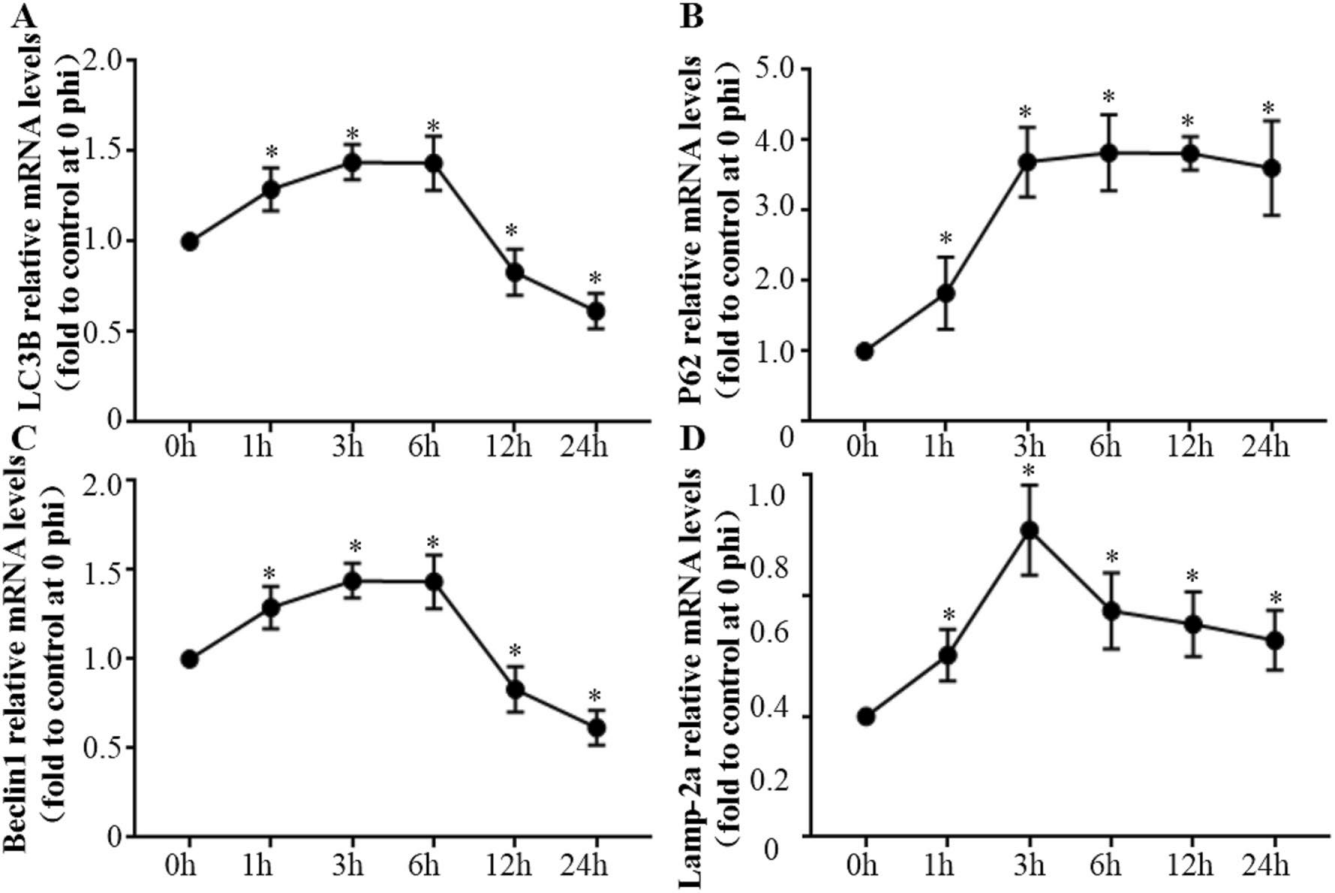
**RT-PCR assays for mRNA expression changes in autophagy-associated genes in bMEC infected by** *M. bovis.* **A** Relative mRNA expression levels of LC3B. **B** Relative mRNA expression levels of P62. **C** Relative mRNA expression levels of Beclin1. **D** Relative mRNA expression levels of Lamp-2a. Data are mean  ±  SD of 3 independent experiments. Standard deviations of individual measurements are indicated as bars. ^*^Compared to the control group (*p * <  0.05).

**Figure 4:**
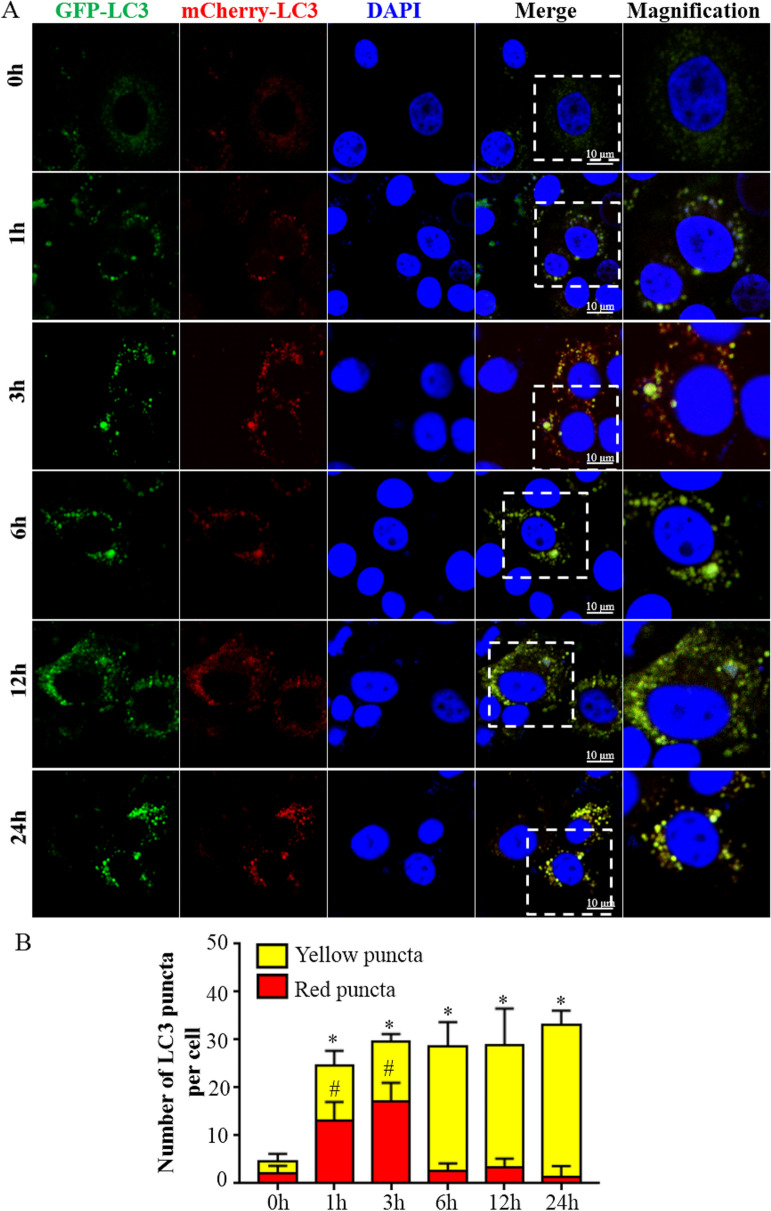
**Laser confocal analysis of autophagy flux assessed by Ad-mCherry-GFP-LC3**. **A** Representative images of mCherry-GFP-LC3 in bMEC. bMEC transfected by Ad-mCherry-GFP-LC3 were infected by *M. bovis*. The autophagy flux was analyzed by a laser confocal method. The mRFP^+^ GFP^−^ LC3 puncta yielded a red signal, whereas the mRFP^+^ GFP^+^ LC3 yielded a yellow signal. **B** Quantification of autophagosomes in bMEC. Twenty cells for each sample and at least 60 cells in each group were used for statistical analyses. SD of 3 independent experiments. Standard deviations of individual measurements are indicated as bars. ^*^Compared to the control group (*p*  <  0.05).

Protein levels of autophagy-associated genes were analyzed by Western blotting. There was increased conversion of LC3I–LC3II from 3 to 12 hpi in infected bMEC compared to control (uninfected) cells (Figure 2A, B; *p*  <  0.05). Levels of P62 protein were decreased in infected cells compared to control cells at 1, 3, and 6 hpi (Figure 2A, C; *p*  <  0.05). Furthermore, Beclin1 was higher in infected cells compared to control cells at 3 and 6 hpi (Figure 2D, E; *p*  <  0.05). Relative mRNA expression levels of autophagy-associated genes were tested by RT-PCR. There were higher levels of mRNA for LC3B and Beclin1 in infected cells compared to control cells at 1, 3 and 6 hpi (Figure 3A, C; *p*  <  0.05). However, mRNA levels of the 2 autophagy indicators were lower in infected versus control cells at 12 and 24 hpi (Figure 3A, C; *p*  <  0.05). The relative mRNA expression levels of P62 and Lamp-2a were increased in infected cells compared to control cells at 1, 3, 6, 12, and 24 hpi (Figure 3B, D; *p*  <  0.05). The autophagy flux was analyzed with an mRFP-GFP-LC3 adenovirus reporter and confocal microscope. Accumulation of LC3 puncta occurred in infected bMEC, whereas there was a diffuse distribution of LC3 in control cells (Figure 4A). Levels of total mRFP^+^ GFP^+^ LC3 puncta (yellow signal) were higher in infected cells at 1 hpi, compared to control cells at 0 hpi (Figure 4B; *p*  <  0.05). Therefore, *M. bovis* induced autophagy in bMEC at 1 and 3 hpi.

### Autophagy flux was blocked

The level of P62 protein underwent a transient decline at 1 hpi (*p*  <  0.05), followed by a time-dependent increase from 3 to 12 hpi (Figures. 2A, C; *p*  <  0.05). However, levels of P62 mRNA increased in infected cells after 1 hpi (Figure 3B; *p*  <  0.05). Decreases in P62 protein levels at 1, 3 and 6 hpi were attributed to autophagic degradation, whereas gradually restored levels of P62 implied that the autophagy flux was blocked at 12 and 24 hpi. Ad-mCherry-GFP-LC3 was used to monitor the autophagic flux in bMEC. The mRFP^+^ GFP^−^ LC3 puncta (red signal), which yielded no or weak GFP signals, increased in infected cells at 1 and 3 hpi, compared to control cells at 0 hpi (Figure 4B; p  <  0.05). However, the subsequent mRFP^+^ GFP^−^ LC3 puncta levels in infected cells at 6, 12 and 24 hpi decreased to levels not significantly different from control cells (Figure 4B). Therefore, autophagosome maturation was inhibited at 6, 12 and 24 hpi. Levels of Beclin1 mRNA and protein increased at 3 and 6 hpi compared to control bMEC (0 h) (*p*  <  0.05), then decreased at 12 and 24 hpi, indicating defective delivery of lysosomal cargo at the later stages (Figures 2D, F,  3C). Overall, there was clear evidence that the autophagy flux was blocked at 12 and 24 hpi in infected bMEC.

### Lysosomes were impaired by *M. bovis* infection

**Figure 5:**
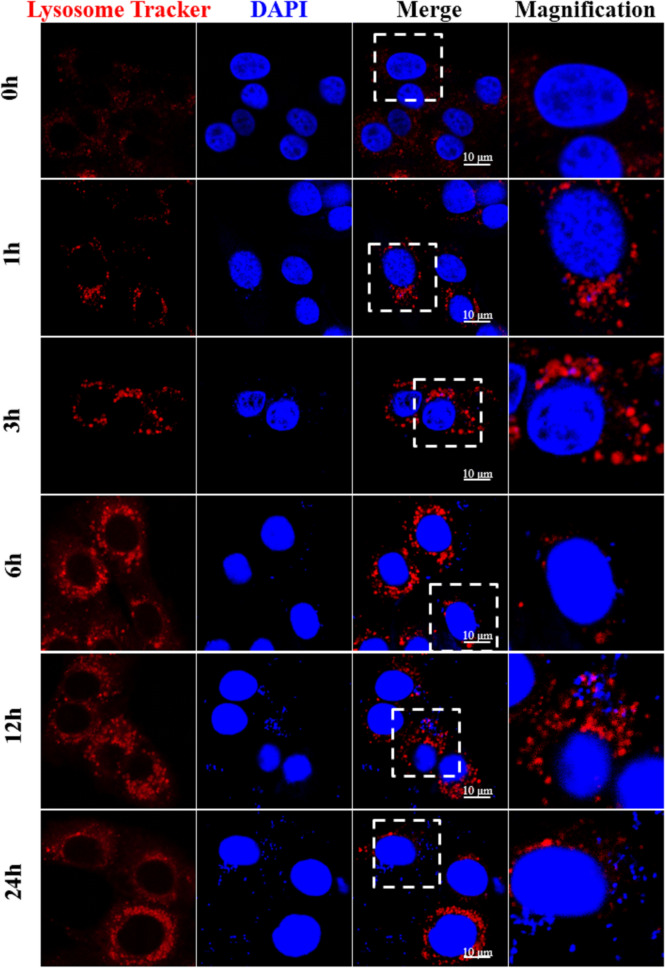
**Lysosomal acidification assays in bMEC infected by** *M. bovis*. Lysosomes in bMEC were stained by Lysotracker Red prior to fixation after infection. Acidified lysosomes were marked by a pH sensitive probe; the red signal indicates acidified lysosomes. Five randomly selected fields per sample were analyzed for each sample and 3 repeats were conducted. A representative field is shown in the figure.

When an autophagy flux is induced, following expansion and closure of the phagopore, an autophagosome matures and forms an autophagolysosome. In many cases, microorganisms internalized by eukaryotic cells are efficiently eliminated in a mature autophagolysosome, which contains lysosomal hydrolases, e.g., lysosomal-associated membrane glycoproteins (LAMP) [5]. These lysosomal hydrolases are responsible for degradation of autophagic substrates. In the present study, levels of Lamp-2a protein were decreased at 6, 12 and 24 hpi compared to cells at 0 hpi (Figure 2D, F; *p * <  0.05). Autophagosome acidification was assessed by fluorescence microscopy. Acidified lysosomes probed by Lyso-Tracker Red yielded a red signal, whereas intracellular DNA particles were stained with DAPI [14]. Based on the evaluation of randomly selected fields, red fluorescence was reduced around intracellular *M. bovis*, indicating that *M. bovis* infection inhibited autophagosome acidification (Figure 5). The results indicate that the lysosomes in bMEC were impaired at 6, 12 and 24 hpi.

### Induction of autophagy promotes maturation of phagosomes, resulting in inhibition of intracellular *M. bovis* replication

**Figure 6:**
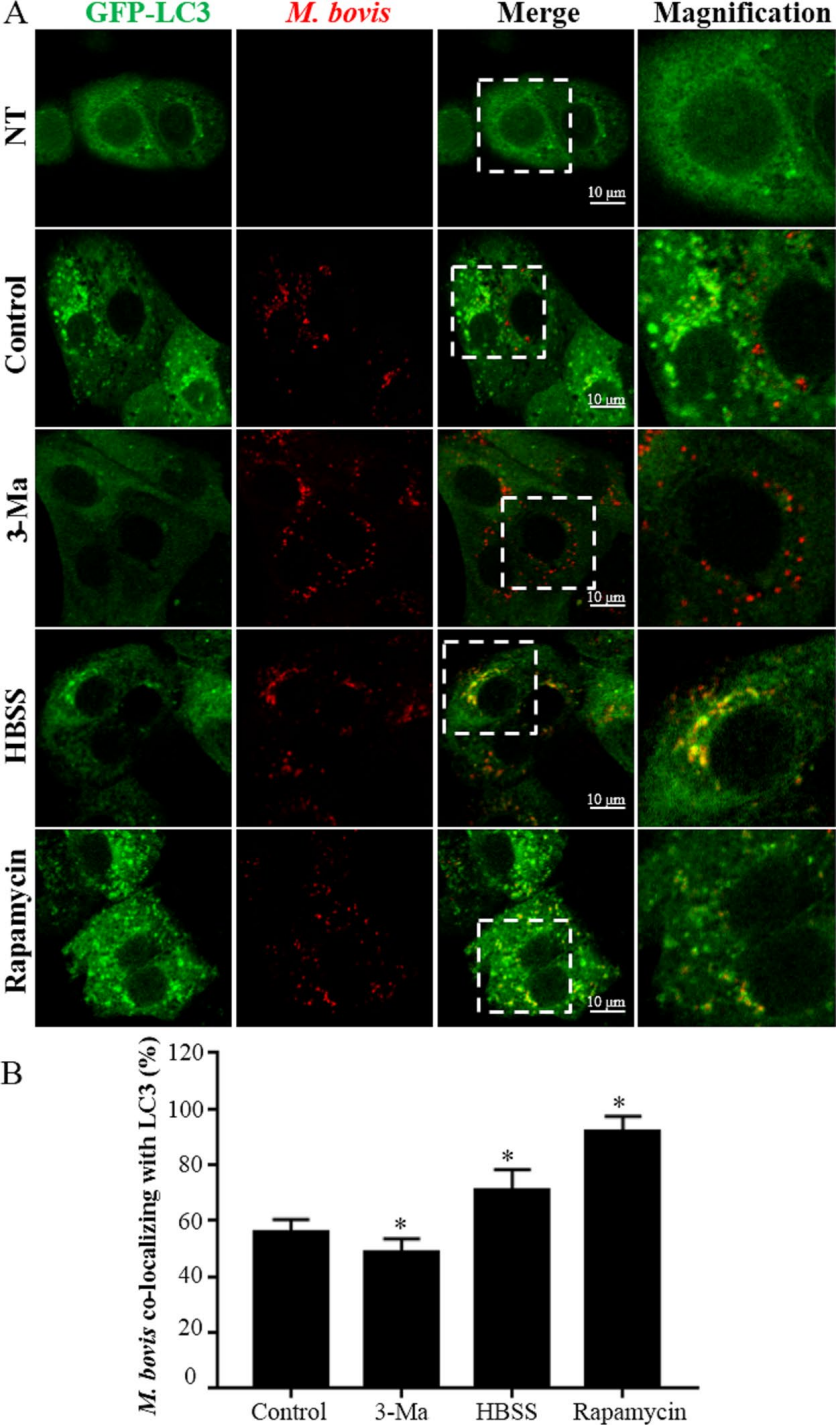
***M. bovis*** translocate into autophagosomes indicated by *M. bovis* co-localizing with GFP-LC3. **A** Representative images of *M. bovis* (Red) co-localizing with GFP-LC3 (Green) at 6 hpi. bMECs transfected by ad-GFP-LC3 was infected by *M. bovis*. Autophagy was enhanced by rapamycin (20 μg/mL) or starvation (Hanks balanced salt solution) after infection. Autophagy was inhibited by pre-incubating bMEC with 5 mM 3-methyladenine in full nutrition medium for 3 h prior to infection. The cells on coverslips were fixed by 4% paraformaldehyde, blocked by 3% with BSA in PBS and permeabilized with 0.2% Triton X-100 in PBS. The *M. bovis* in cells were immunostained with mouse anti-*M. bovis* antibody and Alexa Fluor-conjugated secondary antibody. NT: bMEC without treatment of rapamycin, Hanks balanced salt solution, 3-methyladenine or infection; Control: *M. bovis*-infected bMEC without treatment of rapamycin, HBSS, 3-methyladenine; 3-Ma: *M. bovis*-infected bMEC treated with 3-methyladenine; HBSS: *M. bovis*-infected bMEC treated with Hanks balanced salt solution; Rapamycin: *M. bovis*-infected bMEC treated with rapamycin. **B** Quantification of *M. bovis* co-localizing with GFP-LC3. Images were analyzed quantitatively with Image J software with the JaCoP plugin. Twenty cells for each sample and at least 60 cells in each group were used for statistical analyses. Data are mean  ±  SD of 3 independent experiments. Standard deviations of individual measurements are indicated as bars. ^*^Compared to the control group (*p*  <  0.05).

**Figure 7:**
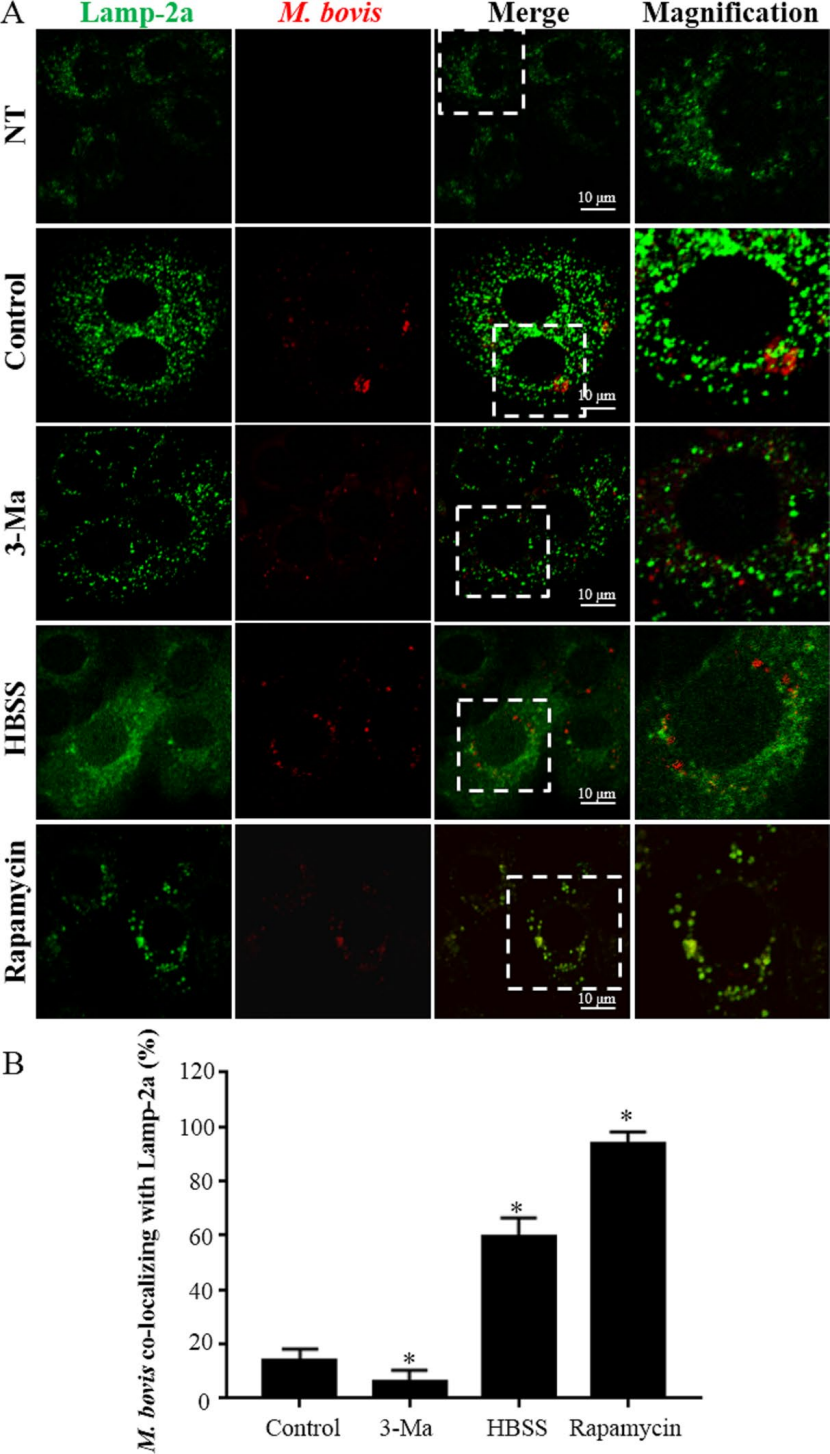
***M. bovis*** translocate into lysosomes, as indicated by *M. bovis* co-localizing with Lamp-2a. **A** Representative images of *M. bovis* (Red) co-localizing with Lamp-2a (Green) at 6 hpi. Autophagy was enhanced by rapamycin (20 μg/mL) or starvation (Hanks balanced salt solution). Autophagy was inhibited by pre-incubating the bMEC with 5 mM 3-methyladenine in full nutrition medium for 3 h prior to infection. NT: bMEC without treatment of rapamycin, Hanks balanced salt solution, 3-methyladenine or infection; Control: *M. bovis*-infected bMEC without treatment of rapamycin, HBSS, 3-methyladenine; 3-Ma: *M. bovis*-infected bMEC treated with 3-methyladenine; HBSS: *M. bovis*-infected bMEC treated with Hanks balanced salt solution; Rapamycin: *M. bovis*-infected bMEC treated with rapamycin. **B** Quantification of *M. bovis* co-localizing with GFP-LC3. Images were analyzed quantitatively with Image J software with the JaCoP plugin. Twenty cells for each sample and at least 60 cells in each group were used for statistical analyses. Data are mean  ±  SD of 3 independent experiments. Standard deviations of individual measurements are indicated as bars. ^*^Compared to the control group (*p * <  0.05).

**Figure 8:**
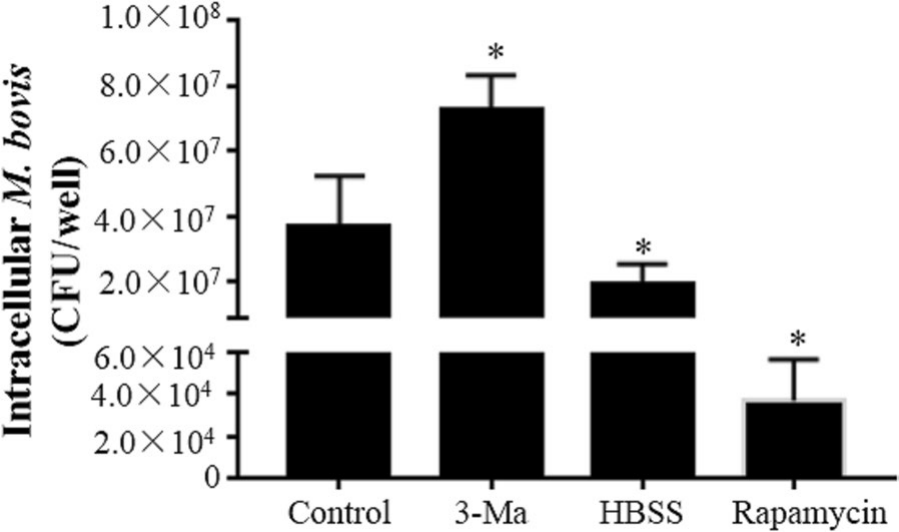
**Intracellular** ***M. bovis*** **assays under various autophagic states.** Intracellular *M. bovis* replication in bMEC under various autophagic states were compared. Control: *M. bovis*-infected bMEC without treatment of rapamycin, HBSS, 3-methyladenine; 3-Ma: *M. bovis*-infected bMEC treated with 3-methyladenine; HBSS: *M. bovis*-infected bMEC treated with Hanks balanced salt solution; Rapamycin: *M. bovis*-infected bMEC treated with rapamycin. Data are mean  ±  SD of 3 independent experiments. Standard deviations of individual measurements are indicated as bars. ^*^Compared to the control group (*p*  <  0.05).

To assess effects of autophagy on intracellular *M. bovis* replication, autophagy in *M. bovis*-infected bMEC was induced by rapamycin or HBSS, or inhibited by 3-Ma. Intracellular *M. bovis* was marked by immunostaining with mouse anti-*M. bovis* serum, prepared as described above. Inhibition of autophagosomes was apparent at 6 hpi; therefore, samples were collected at 6 hpi and cells were sequentially assessed with fluorescence microscopy. Images were analyzed with Image J software with the JaCoP plugin. Co-localization of LC3 and *M. bovis* indicate that the LC3-positive *M. bovis* proportions were higher in HBSS or rapamycin-treated cells compared to the control cells (infected with *M. bovis* but not treated with rapamycin, HBSS or 3-Ma; Figure 6; *p*  <  0.05), but were lower in 3-Ma treated cells (Figure 6; *p*  <  0.05). Subsequently, co-localization of Lamp-2a and *M. bovis* were assessed by fluorescence microscopy and intracellular *M. bovis* enumerated. Both rapamycin and HBSS significantly increased the Lamp-2a positive *M. bovis* proportions in bMEC compared to the control cells (*p*  <  0.05), whereas it was decreased by 3-Ma (Figure 7; *p*  <  0.05). Non-treated cells (NT, bMECs were not treated with rapamycin, HBSS, 3-Ma or infection) had low levels of red signal, indicating the good specificity of the immunostaining methods by using the mouse anti-*M. bovis* serum (Figs. 6A, 7A). The cells at 24 hpi were collected as samples for enumerating intracellular *M. bovis*. The intracellular *M. bovis* load was significantly decreased in cells treated with rapamycin or HBSS, whereas it was increased by 3-Ma (Figure 8; *p*  <  0.05). Effects of rapamycin and 3-Ma on *M. bovis* viability were tested. These 2 compounds did not decrease viability of *M. bovis* in PPLO medium (Additional file 2A; *p*  <  0.05). Similarly, the effects of rapamycin and 3-Ma on bMEC viability were also tested; neither of these 2 compounds decreased viability of bMEC in DMEM containing 10% FBS (Additional file 2B; *p*  <  0.05).

## Discussion

Autophagy is not only a conserved catabolic pathway for recycling intracellular materials, but also a defensive strategy used by eukaryotic cells to degrade intracellular pathogens such as *Staphylococcus aureus* and *M. tuberculosis.* In contrast, there is emerging evidence that autophagy enables some bacteria (e.g., *Salmonella*) to replicate in host cells [5]. However, there are limited data regarding autophagy during infections with *Mycoplasma* species, the smallest self-replicating microorganisms. In this study, *M. bovis* and bMEC were used to establish an in vitro model to analyze effects of autophagy on non-specialized phagocytes on the intracellular replication of mycoplasma. This study was the first to document that: (1) *M. bovis* induced autophagy in a nonprofessional phagocytic cell; (2) the autophagy flux was subsequently blocked by *M. bovis*; and (3) *M. bovis* subverted the autophagosome to promote intracellular replication in bMEC. Therefore, our hypothesis that *M. bovis* subverts autophagy to promote its intracellular replication was supported.

An in vitro infection model with *M. bovis* and primary bMEC was established in this study. *M. bovis* can invade many types of cells, including epithelial cells and lymphocytes [18–20]. Therefore, this pathogen can effectively evade humoral immunity and bactericidal effects of certain antibiotics, enabling it to persist in the host [18]. Adhesion and invasion of *M. bovis* are crucial to successful infection. Furthermore, it was speculated that *M. bovis* invasion into epithelial cells occurs mainly through endocytosis [18]. In our previous study, adhesion and invasion of *M. bovi*s were verified with an infection model using MAC-T cells, a bovine mammary gland epithelial cell line [17]. However, effects of autophagy on intracellular *M. bovis* were not fully elucidated. In this study, primary bMEC were used. The purity of bMEC was analyzed by staining with CK18, a marker of luminal epithelial cells [24, 25]. The purities of the bMEC in passage 6–9 were all over 95%. The ability of *M. bovis* to adhere to the surface of bMEC and subsequently become intracellular was confirmed by TEM. Moreover, intracellular *M. bovis* was present in a membrane-like structure in the cytoplasm of bMEC. Hence, our in vitro infection model was successful. Based on slow-growth kinetics of *M. bovis* during the first 9 h of infection, we inferred that *M. bovis* replication was initially restricted. However, thereafter, numbers of intracellular *M. bovis* increased significantly. Thus, *M. bovis* survival and replication were restricted during the initial 9 h of infection in bMEC, but *M. bovis* replicated significantly thereafter, implying changes that produced a more favorable growth environment.

In this study, *M. bovis* activated autophagy in bMEC at early stages of infection. To avoid host autophagic defenses, pathogenic species have developed 3 strategies: evasion, inhibition, and subversion [5, 9]. For example, a type III secretion system enables *Shigella* to avoid being detected by the host autophagic machinery [12]; *Legionella pneumophila* inhibits autophagy in mammalian cells [30]; and *Staphylococcus aureus* impairs autophagy for replication [28, 31]. Similarly, *Mycoplasma pneumoniae* induced autophagy in mice [14], whereas *Mycoplasma ovipneumoniae* activated autophagy in RAW 264.7 cells [13]. In this study, to investigate autophagy, the ultramicrostructure of intracellular *M. bovis* was investigated. There were visible membranes surrounding the *M. bovis* with cytoplasmic (membranous) material that may have resulted from intraluminal vesicle formation or from fusion with autophagic vesicles, consistent with previous studies of *Staphylococcus aureus* targeted by autophagy [27]. Secondly, increased turnover of LC3II (the most widely recognized molecular indicator of autophagy) and a transient decrease in P62 was induced by *M. bovis* at 1 hpi. Therefore, in the present study, *M. bovis* induced autophagy in bMEC when adhesion and invasion were successfully established.

However, the autophagic flux was subsequently blocked by *M. bovis* infection at later stages. Autophagic flux refers to the entire process of autophagy, including delivery of cargo to lysosomes and its subsequent breakdown [32, 33]. To detect the autophagic flux in bMEC, P62 was analyzed by Western blotting and RT-PCR, followed by detection of LC3 puncta with fluorescence microscopy. Under normal circumstances, P62 is a link between LC3 and substrates, with P62 and P62-bound substrates incorporated into the completed autophagosome during the initial stage and subsequently degraded in autolysosomes. Therefore, increasing P62 protein levels are indicative of inhibition of autophagy flux [26, 34]. In this study, P62 protein levels decreased significantly during the early stage of infection (1 hpi), indicating activation of autophagy. However, continuous increases in expression of P62 mRNA and gradually restored P62 protein levels in bMEC indicate that the degradation of P62 protein was inhibited at 12 and 24 hpi. The mRFP/mCherry-GFP method enabled concurrent assessment of both the induction of autophagy and the subsequent flux through autophagic compartments under native conditions [26]. The mRFP^+^ GFP^−^ LC3 puncta increased significantly in infected cells at 1 and 3 hpi, indicating that autophagosome maturation was activated. However, subsequent decreases in mRFP^+^ GFP^−^ LC3 puncta levels in infected cells at 6, 12 and 24 hpi indicate that autophagosome maturation was inhibited. Therefore, in bMEC infected with *M. bovis*, the autophagy flux was blocked at 12 and 24 hpi.

Maturation of the autophagosome was subverted in bMEC. An autophagosome is the core functional center for degradation of autophagy-targeted substrates [35, 36]. There is emerging evidence that some bacteria, e.g., *Staphylococcus aureus* and *M. tuberculosis*, induce defects in phagosome acidification [11, 31]. In this study, levels of Beclin1 protein increased at 3 and 6 hpi, but subsequently decreased at 12 and 24 hpi, indicating a defect in delivery of lysosomal cargo. In addition, Lamp-2 protein levels decreased significantly at 6, 12 and 24 hpi, providing further evidence that lysosomal function was impaired by *M. bovis* infection.

Autophagy is an antibacterial defense [26]. For example, activated autophagy results in the reduced intracellular *Mycoplasma ovipneumoniae* in macrophage [13]. To confirm the relationship of autophagy and intracellular *M. bovis*, bMEC were treated by starvation, rapamycin or 3-Ma. In this study, intracellular numbers of *M. bovis* were inversely associated with the level of autophagy in host cells, consistent with previous reports for *Mycoplasma ovipneumoniae* [13]. Moreover, both starvation and rapamycin increased the LC3 positive *M. bovis* proportions and the Lamp-2a positive *M. bovis* proportions, and decreased intracellular *M. bovis* replication in bMEC, whereas 3-Ma yielded opposite results. Therefore, we inferred that intracellular replication of *M. bovis* was regulated by autophagy, and under typical circumstances, it was promoted by blocking the delivery of *M. bovis* to the autophagosome and lysosome. Furthermore, activation of autophagy counteracted the *M. bovis*-induced blockade of phagosome maturation in bMEC. The outcome was similar to the *M. tuberculosis* variant *bovis* BCG, whereas *Staphylococcus aureus* had increasing loading of bacteria when autophagy was enhanced [5, 37]. An important limitation is that this study used an in vitro model that assessed the fate of intracellular *M. bovis* in bMEC, a non-specialized phagocyte. In contrast, under in vivo conditions, there would be more complex mechanisms, including host–pathogen interactions for other types of cells, including specialized phagocytes or immune cells. Host-Directed Therapy, based on modulation of autophagy, is a potential mechanism for effective clearance of infection [38]. Autophagy is a novel model to study intracellular biochemical mechanisms. Furthermore, modulation of autophagy following intracellular *M bovis* infections provided insights into regulation of autophagy and indications of how *M. bovis* may induce persistent infections.

In conclusion, *M. bovis* infection induced autophagy in bMEC, but the ensuing autophagy flux was subsequently impaired by inhibiting maturation of autophagosomes. *M. bovis* subverted autophagy to promote its replication in bMEC. These findings provide new knowledge and the impetus for additional studies to elucidate interactions between *M. bovis* and host cells. Perhaps in the future we can regulate autophagy to develop innovative therapies and perhaps improved vaccines.

## Supplementary Information

**Additional file 1 Identification of bMEC by staining with cytokeratin 18. A **Representative images of randomly selected fields in bMEC from passages 6–9; bMEC were stained by cytokeratin 18 (CK18) and their nuclei were stained by DAPI; Arrows point to CK18 negative cells; **B** quantitative analysis of the bMEC from passages 6–9; purity of bMEC was defined as the proportion of CK18 positive cells among total cells (stained by DAPI). Data are mean ± SD of 3 independent experiments. Standard deviations of individual measurements are indicated as bars.

**Additional file 2 Effects of rapamycin and 3-methyladenine on M. bovis or bMEC. A** Effects of rapamycin and 3-methyladenine on *M. bovis* viability in PPLO medium. **B** Effects of rapamycin and 3-methyladenine on viability of bMEC in DMEM medium containing 10% FBS by CCK-8 assays. Data are mean ± SD of 3 independent experiments. Standard deviations of individual measurements are indicated as bars. ^*^Compared to the control group (*P *< 0.05).

## Data Availability

All data generated or analyzed during this study are included in this published article.

## Abbreviations

bMEC: bovine mammary epithelial cells
MOI: multiplicity of infection
hpi: hours post-infection
HBSS: Hanks balanced salt solution
PFA: paraformaldehyde
BSA: bull serum albumin
3-Ma: 3-Methyladenine
FBS: fetal bovine serum
GM: gentamicin
PBS: phosphate-buffered saline
LAMP: lysosomal-associated membrane glycoproteins
TEM: transmission electron microscopy
