# Supplementary figures and images for: Mycoplasma bovis subverts autophagy to promote intracellular replication in bovine mammary epithelial cells cultured in vitro
Source: Vet Res. 2021 Oct 14;52:130. doi: 10.1186/s13567-021-01002-z (PMC8515657; doi:10.1186/s13567-021-01002-z)

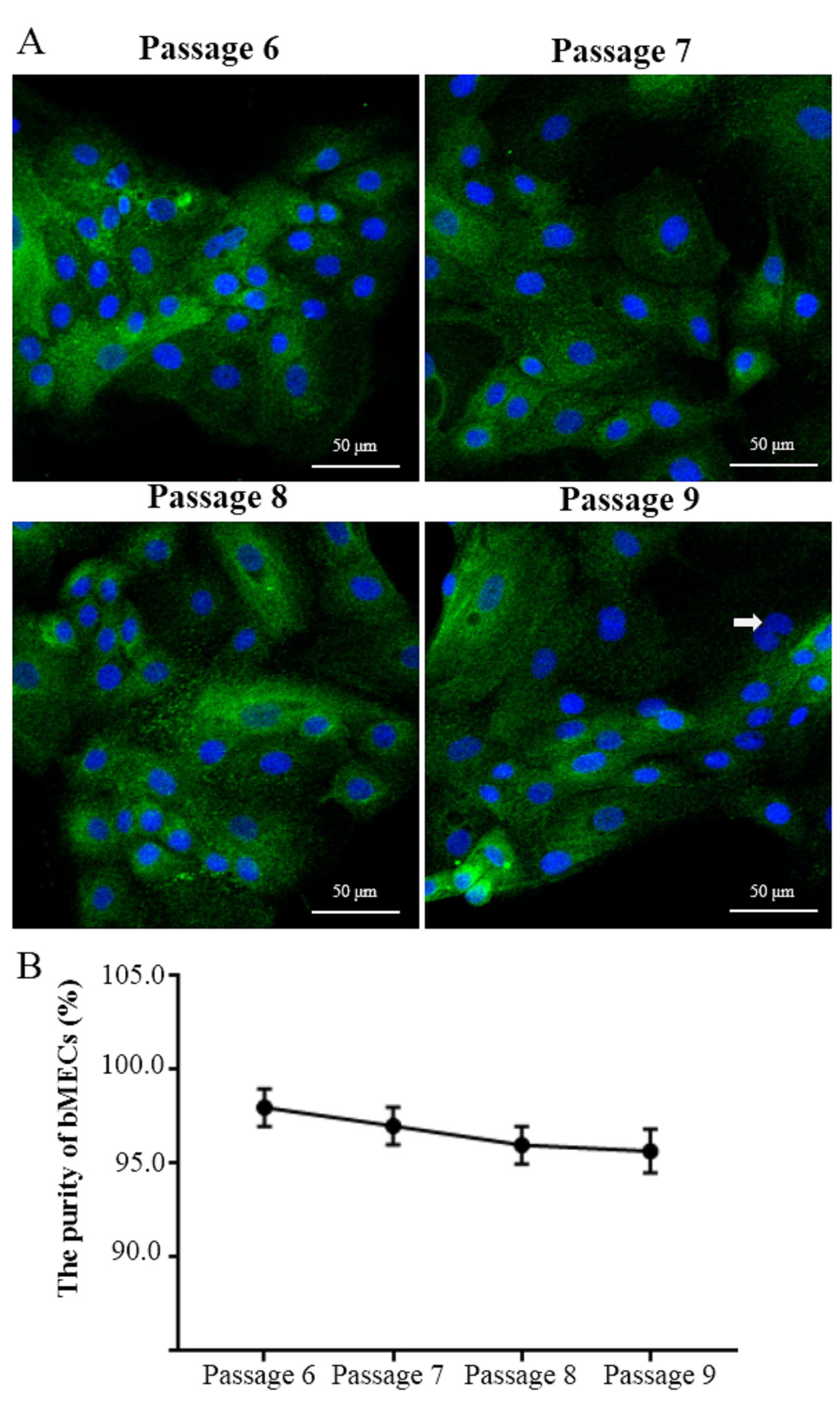

Supplement: Supplementary file 1 — Additional file 1 Identification of bMEC by staining with cytokeratin 18. A Representative images of randomly selected fields in bMEC from passages 6–9; bMEC were stained by cytokeratin 18 (CK18) and their nuclei were stained by DAPI; Arrows point to CK18 negative cells; B quantitative analysis of the bMEC from passages 6–9; purity of bMEC was defined as the proportion of CK18 positive cells among total cells (stained by DAPI). Data are mean ± SD of 3 independent experiments. Standard deviations of individual measurements are indicated as bars. [file 13567_2021_1002_MOESM1_ESM.tif]

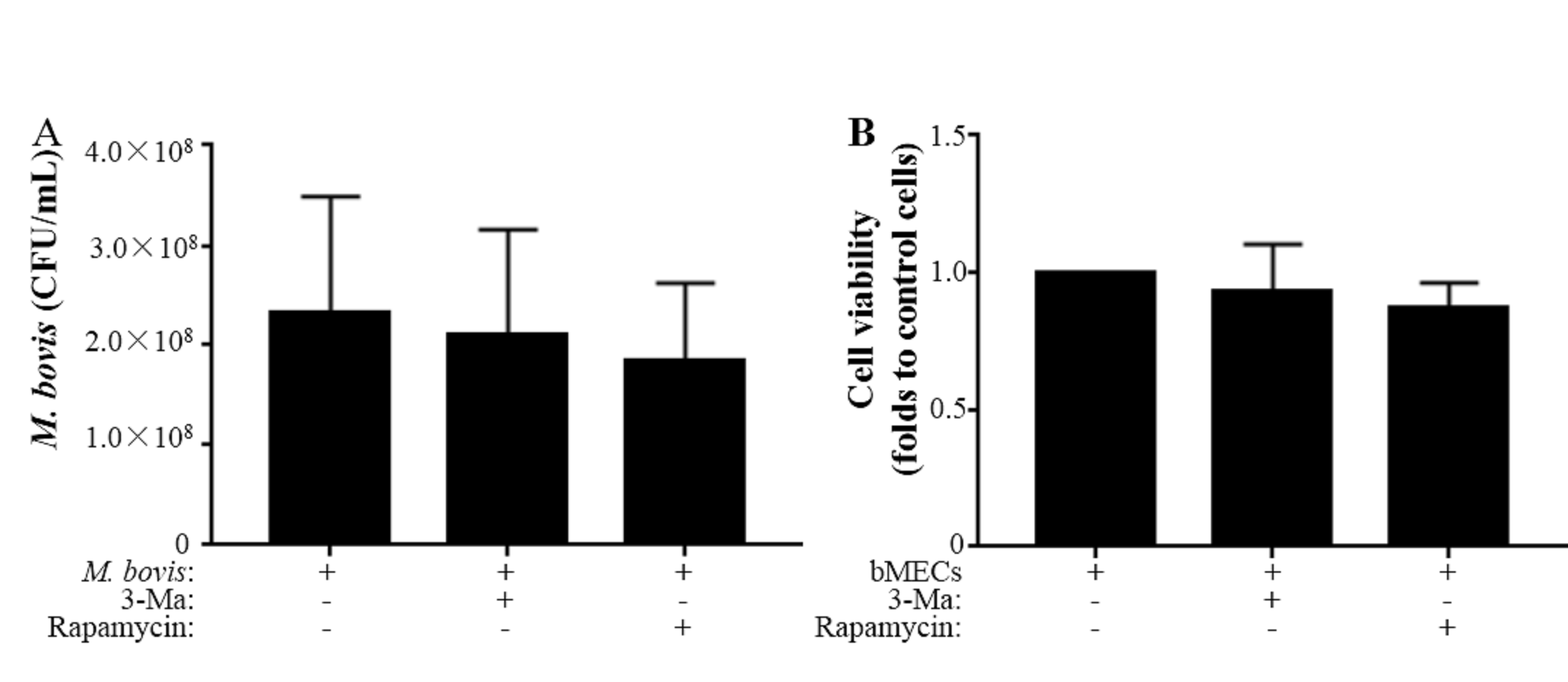

Supplement: Supplementary file 2 — Additional file 2 Effects of rapamycin and 3-methyladenine on M. bovis or bMEC. A Effects of rapamycin and 3-methyladenine on M. bovis viability in PPLO medium. B Effects of rapamycin and 3-methyladenine on viability of bMEC in DMEM medium containing 10% FBS by CCK-8 assays. Data are mean ± SD of 3 independent experiments. Standard deviations of individual measurements are indicated as bars. *Compared to the control group (P < 0.05). [file 13567_2021_1002_MOESM2_ESM.tif]
